# Experimental Study of a Compact Microwave Applicator for Evaporation of Airflow-Entrained Droplets

**DOI:** 10.3390/ma15196765

**Published:** 2022-09-29

**Authors:** Jesus Nain Camacho Hernandez, Guido Link, Markus Schubert, Uwe Hampel

**Affiliations:** 1 Institute for Pulsed Power and Microwave Technology IHM, Karlsruhe Institute of Technology, Hermann-von-Helmholtz-Platz 1, 76344 Eggenstein-Leopoldshafen, Germany; 2 Institute of Fluid Dynamics, Helmholtz-Zentrum Dresden-Rossendorf, Bautzner Landstr. 400, 01328 Dresden, Germany; 3 Institute of Process Engineering and Environmental Technology, Technische Universität Dresden, Helmholtzstr. 14, 01069 Dresden, Germany; 4 Chair of Imaging Techniques in Energy and Process Engineering, Technische Universität Dresden, 01062 Dresden, Germany

**Keywords:** droplet removal, open-cell foams, microwave heating, evaporation

## Abstract

In many energy and process engineering systems where fluids are processed, droplet-laden gas flows may occur. As droplets are often detrimental to the system’s operation, they need to be removed. Compact engineering solutions for the removal of entrained droplets are difficult to achieve with conventional flow control and heat transfer approaches and thus droplet removal devices are hence often costly and bulky. In this study, we analyzed the potential of a compact technology based on droplet capture and in situ evaporation by microwave heating. For that, we designed a microwave applicator containing a porous droplet separator for capturing and evaporating droplets. The application of open-cell ceramic foams as filter medium reduced 99.9% of the volumetric flow of droplets, while additional microwave exposure increases reduction to 99.99%. In addition, microwave-heated foams prevent droplet re-entrainment and structure-borne liquid accumulation within foams, thus avoiding water clogging and flooding.

## 1. Introduction

Droplet-laden gas streams pose a risk to industrial processes involving fluids and moving parts, such as in turbine blades where erosion decreases their efficiency and safety [1,2]; therefore, effective removal of droplets from the process streams is essential.

The separation of gas-borne liquid droplets is today mainly based on mechanical separation. According to the recent review on droplet removal [3], separation is accomplished through four mechanisms, i.e., direct interception, inertial impaction, diffusional deposition and gravitation settling. Whether a certain mechanism takes place depends on the droplet substance and size, which defines the magnitude of the droplet momentum opposing the drag forces exerted by the gas flow. At a condition known as entrainment, the droplet separation is particularly complicated since the droplets are embedded into the gas following the same streamline pattern.

Droplet removal equipment separates droplets in two steps [4,5]. In the primary separation (first step), inertia forces act upon the droplets while they are subject to changes in the flow direction. Since the droplets cannot follow the bent streamlines, they end up colliding with the separator walls and are deposited. In the secondary separation (second step), droplets that are deposited (primary droplets) eventually coalescence with others to form a liquid film that is conducted to a draining section for its discharge. At the primary separation, droplet impingement can produce splashing. Splashes arise when primary droplets expand in a rim of fluid that sprays smaller droplets (secondary droplets or satellite droplets), which are then re-entrained into the gas stream. Studies on splashing [6,7,8,9] have shown that droplet–wall interactions (deposition or splashing) depend on impaction regime and surface morphology. Moreover, continuous wetting of the structure results in structure-borne liquid accumulations that cannot be effectively removed, also leading to secondary droplet production caused by the gas shear forces on the liquid film. Moreover, in severe cases, the discharge capacity of the droplet separators is exceeded and results in flooding [10]. Usually, this is counteracted by the sequential arrangement of several separators, however, at the expense of higher pressure drop.

Evaporation of droplets is another way to reduce the droplet load. The main advantage is the avoidance of liquid accumulation, as the liquid is evaporated rather than collected for drainage. However, high energy consumption and equipment costs limit its application to a downstream stage after mechanical separation. Examples of equipment using post-evaporation are moisture separator reheaters, which are used to evaporate droplets in steam flows. Commonly, they incorporate a coarse droplet separator (i.e., vanes, corrugated plates) and various stages of heat exchanger tubes [11]. The heat provided by the tubes is intended to evaporate the liquid rather than the entrained droplets, since very long ducts and high overheating would be required to transfer enough energy for complete evaporation of very fast-moving droplets (short residence time).

One more aspect to consider is that the series of droplet separators and subsequent evaporation equipment requires bulky equipment and recirculation lines, which can hardly be retrofitted into steam conducts and which hampers revamping existing industrial facilities. Alternatively, simultaneous application of mechanical droplet separation and in situ evaporation would provide compact solutions for facilities that overcome structure-borne liquid accumulations in fine droplet separators. However, heating such fine separators (i.e., mist eliminators, wire/knit meshes) is inefficient due to the high thermal resistance of their thin solid structures. In addition, Joule heating requires a well-defined and homogeneous electrical resistance to ensure uniform heating, which is technically challenging to apply in fine separators. Alternatively, volumetric heating using microwaves could overcome these challenges. Microwave technology has been studied and implemented over the past decades to intensify existing commercial processes, owing to its short start-up, selective heating and the ability of microwaves to propagate through solid media [12,13]. Furthermore, extensive research and development in recent years has reduced the capital cost of the technology and increased the safety of microwave systems. A disadvantage of using conventional droplet separators along with microwave heating is potential arcing, which may especially happen at sharp metallic elements of current fine separators.

In this work, we propose a novel approach for droplet removal consisting of droplet capture and in situ droplet evaporation by using microwave-heated open-cell solid foams. Open-cell solid foams with porosities (ratio of the volume of voids to the total volume of the foam) ranging from 0.8 to 0.95 provide a network of thermally conductive materials with hydraulically large accessible void volumes that promote fine droplet separation. For this purpose, we designed and constructed a microwave applicator. The approach is evaluated for droplet removal in saturated airflows. Airflows with velocities ranging from 2.5 to 14 ${\mathrm{ms}}^{-1}$, maximum droplet sizes up to 250 µm and droplet volume fractions below 0.1 were used as study targets.

## 2. Materials and Methods

### 2.1. Open-Cell Ceramic Foams

Among the various open-cell ceramic foams that could serve as droplet separators, foams based on silicon carbide (SiC) exhibit considerably good characteristics, such as relative uniform pore densities, high heat conductivities, moderate microwave heating susceptibilities, and low pressure drop [14,15]. Their high-temperature stability, hardness, and corrosion resistance makes these foams even more an outstanding candidate for droplet evaporation. Therefore, the SiC-based open-cell foams—silica-bonded silicon carbide (SBSiC), silicon-infiltrated silicon carbide (SiSiC) and pressureless sintered silicon carbide (SSiC)—have been selected for the study. Two cylindrical samples (diameter = 54.2 mm and height = 70 mm) per linear pore densities of 30 ppi (pores per inch), 45 ppi and 60 ppi with porosities P between 86.8% and 90.6% (see Table 1, reporting average porosity of each pore density samples) were manufactured (IKTS Fraunhofer, Germany).

### 2.2. Pressure Drop and Droplet Residence Time

Entrained droplets move with a terminal droplet velocity $u_{d}$ equal or very close to the gas phase velocity. Droplets experience a reduction in their velocity as they move within the open-cell foams due to increased droplet–droplet and droplet–walls interactions such as impingement, which lead to deposition, coalescence, breakup, splash and re-entrainment phenomena. Eventually, the number of inlet gas-borne droplets decreases because of significant deposition on the foam walls, and those droplets that manage to pass through are slowed. Moreover, secondary droplets generated on dry surfaces and re-entrained are not larger than the primary droplets leaving the foams. One way to describe the droplet passage through the foams is based on their residence time; i.e., droplets are quantified in terms of the frequency distribution.

Pressure drop and droplet residence time depend on foam’s skeletal structures. Since the SBSiC, SiSiC and SSiC foam samples were produced by replication technique using the same polymeric template, their skeletal structure is similar and they feature common pressure drop and droplet residence time. The pressure drop of the SiC-based foams $\nabla p_{\mathrm{foam}}$ is well correlated by the modified Ergun equation [15]

(1) $$\nabla p_{\mathrm{foam}}=\left(559.6{\left(1-P\right)}^{2}S_{\mathrm{foam}}^{2}\mu_{g}u_{g}+2.5\left(1-P\right)S_{\mathrm{foam}}\rho_{g}\left|u_{g}\right|u_{g}\right)P^{-3},$$

where $S_{\mathrm{foam}}$ is the specific surface area, $\mu_{g}$ is the gas dynamic viscosity, $\rho_{g}$ is the gas density and $u_{g}$ is the gas phase velocity normal to the flow displacement. The residence time of droplets Λ was taken from a previous study [15] and fitted with a modified Dagum distribution as

(2) $$\Lambda \left(t\right)\left(t;k,\alpha ,\beta ,\gamma ,\zeta \right)=\alpha k{\left(\frac{\left(t-\gamma \right)}{\beta }\right)}^{\alpha \zeta -1}/\left[\beta {\left(1+{\left(\frac{\left(t-\gamma \right)}{\beta }\right)}^{\alpha }\right)}^{k+1}\right].$$

The values of the fitting parameters k,α,β,γ and ζ were reported recently [15].

### 2.3. Dielectric Properties

**Figure 1:**
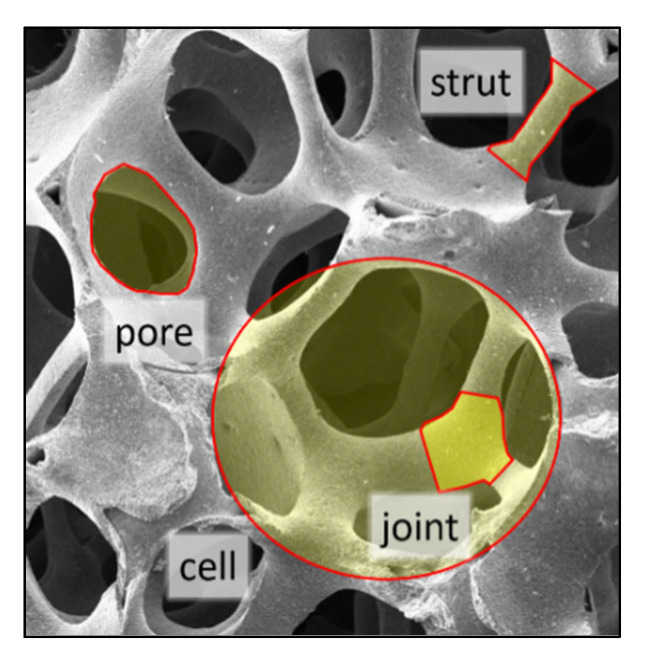
Internal structural elements of open-cell foams.

The development of a suitable microwave applicator requires permittivity data of the open-cell ceramic foams. The permittivity is a complex-valued quantity $\varepsilon =\varepsilon^{\prime }-j\varepsilon^{\prime \prime }$, constituted by the dielectric constant $\varepsilon^{\prime }$ and loss factor $\varepsilon^{\prime \prime }$. Macroscopically, the permittivity of foams can be considered as that of a homogeneous material, referred to as an effective medium and exhibits an effective permittivity $\varepsilon_{\mathrm{eff}}$. That holds as long as the internal structural elements (e.g., pores, cells, struts and joints, as illustrated in Figure 1) are much smaller than the wavelength of the electromagnetic wave propagating through the material.

The effective permittivity of open-cell foams can be well described using Platonic solids as building elements of the open-cell skeletal structures [16], so called Platonic foams. The relation for predicting $\varepsilon_{\mathrm{eff}}$ based on dodecahedrons is

(3) εeff=−2P(1+P2)(εs−εf)+εf[(Re(εsεf)qm′+q0′)+j(Im(εsεf)qm″+q0″)]+εs,

where $\varepsilon_{s}$ and $\varepsilon_{f}$ are the permittivity of skeleton material and void filling medium (i.e., droplet-laden gas flow), respectively. The parameters $q_{0}^{’},\;q_{0}^{’’},\;q_{m}^{’}\;\mathrm{and}\;q_{m}^{’’}$ are summarized elsewhere [16]. The later relation was used for calculating $\varepsilon_{s}$ of the SiC-based foams by using $\varepsilon_{\mathrm{eff}}$ determined from previous experiments [17].

The filling medium is the droplets surrounded by the gas phase. As the maximum moisture content considered is 10% (i.e., droplet volume fraction of 0.1), which is a common value for many industrial exhausts [11]. Accordingly, the filling medium can be treated as an effective medium. Assuming that droplets are spherical and homogeneously dispersed in the air, the Maxwell–Garnett mixing relation (Maxwell-type relation, nonsymmetric) provides good estimates of $\varepsilon_{f}$ for the droplet-laden or wet steam [18]. The Maxwell–Garnett relation is

(4) $$\frac{\varepsilon_{f}-\varepsilon_{g}}{\varepsilon_{f}+2\varepsilon_{g}}=\delta_{d}\left(\frac{\varepsilon_{d}-\varepsilon_{g}}{\varepsilon_{d}+2\varepsilon_{g}}\right)\;,$$

where $\varepsilon_{d}$, $\varepsilon_{g}\;$and $\delta_{d}$ are the permittivities of droplets and gas, and droplet volume fraction, respectively. Note that the droplet volume fraction corresponds only to that of the fluid phase in the stream, so that $\delta_{d}+\delta_{g}=1$. The dielectric permittivity of the droplets is equal that of water, which can be described by its rotational molecular relaxation time using the Debye formula

(5) $$\varepsilon_{d}=\varepsilon_{\infty }\left(T\right)+\frac{\varepsilon_{\mathrm{st}}\left(T\right)-\varepsilon_{\infty }\left(T\right)}{1-i\omega \tau \left(T\right)},$$

where $\varepsilon_{\mathrm{st}}$ and $\varepsilon_{\infty }$ are static (at zero frequency) and optical frequency (at infinite frequency) permittivities, respectively, depending on the temperature *T*, ω is the angular frequency and τ is the rotational relaxation time. The parameters τ, $\varepsilon_{\mathrm{st}}$ and $\varepsilon_{\infty }$ for a temperature ranging from 0 to 100 °C at 1 atm can be found elsewhere [19]. The Debye formula is limited to 1 THz (covering the microwave frequency range).

**Figure 2:**
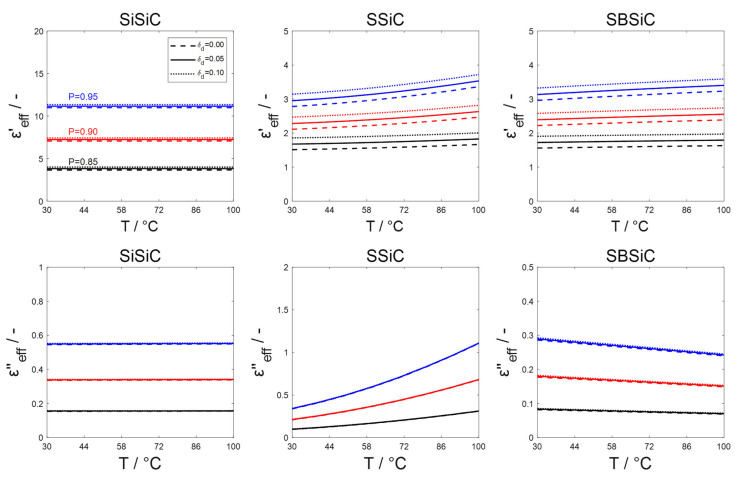
Effective permittivity of the system consisting of foam skeleton, air/steam and dispersed water droplets estimated based on the average skeleton permittivity (notation shown in the upper left subfigure holds for all subfigures).

As the droplets evaporate, it would be necessary to consider the influence of the water vapor dielectric properties on $\varepsilon_{f}$. Yet, the dielectric constant of saturated water vapor of ~1.006 (T=373 K and atmospheric pressure) [20,21] is much lower than that of saturated liquid water, which is higher than 55 [20,21] (due to its much lower density), such a significant difference is also expected for the loss factor [22]. The water vapor content and permittivity are negligible compared to the effective permittivity and volume fraction of SiC-based foams; thus, it is not necessary to differentiate between water vapor and air permittivities in Equation (3). The evaporation of droplets decreases $\delta_{d}$. The effective permittivity for foam porosities of 0.85, 0.90 and 0.95, and droplet volume fractions of 0.00, 0.05 and 0.10 at a microwave frequency of 2.45 GHz calculated using Equations (3)–(5) is illustrated in Figure 2.

Considering the foam as an effective material, the size of the inclusions must not exceed the maximum inclusion diameter $d_{\mathrm{icl}}$ calculated in accordance with the inclusion-size parameter X [16,23] as

(6) $$d_{\mathrm{icl}}=\lambda_{\mathrm{inc}}X/\left(\pi \sqrt{\varepsilon_{\mathrm{eff}}^{’}}\right),$$

where $\lambda_{\mathrm{inc}}$ is the wavelength of the incident microwave radiation. Using the threshold value X=0.15 as suggested by Mishchenko et al. [23] along with the maximum value of the foam’s $\varepsilon_{\mathrm{eff}}^{’}$ (P=0.85, T=100 °C, $\delta_{d}=0.1$), the allowed maximum $d_{\mathrm{icl}}$ is 1.7 mm (SiSiC), 3.2 mm (SBSiC) and 3.1 mm (SSiC), which is above the largest cell size (at minimum pore density) of the foams corresponding to 1.36 ± 0.17 mm (SiSiC foam, 30 ppi). Note that only the average cell diameter was measured for SiSiC. However, due to the similarity with the skeleton of foams, their cell size is expected to be similar.

### 2.4. Governing Equations

A microwave applicator is essentially composed of a microwave source and a transmission line, such as waveguide and a cavity, respectively. The test material (i.e., solid foams in this work) is placed inside the cavity. Typical cavities comprise cylindrical or rectangular waveguides, which are closed or shortened at one end to reflect the incident microwave. For designing the applicator for droplet-laden steam evaporation using microwave-heated foams, it was necessary to examine electric field, temperature distribution and flow profile within the applicator. As mentioned in Section 3.2, an effective medium treatment for the dielectric properties of the mixture (gaseous medium, foam skeleton and droplets) was adopted in the numerical calculations.

The thermal energy generated from the microwaves $Q_{\mathrm{MW}}$ is directly proportional to the square of the *E*-field strength, that is

(7) $$Q_{\mathrm{MW}}=\omega \varepsilon_{\mathrm{eff}}^{\prime \prime }\varepsilon_{0}\int_{V}^{.}E^{2}dV,$$

where E is the electric field, ω is the angular frequency and V is the considered volume.

As the droplet-laden stream is a very dilute flow, its profile can be analyzed as a single-phase flow using the equations of continuity and conservation of inertial momentum, given as

(8) ∇·(ρu)=0

(9) $$\frac{Du}{Dt}=-\frac{\nabla p}{\rho_{g}}+\frac{1}{\rho_{g}}\nabla \times \bar{\tau }+g\;$$

where D/Dt is the Lagrangian time derivative, $\bar{\tau }$ is the summation of viscous stress tensor and turbulence stress, $\rho_{g}$ is the gas density and g is the gravity acceleration constant. While the continuity equation does not change for incompressible flows in the porous medium, the conservation of momentum equation does according to

(10) $$\frac{1}{P}\left(\frac{\partial u}{\partial t}+\frac{1}{P}\left(u\cdot \nabla \right)u\right)=-\frac{\nabla p}{\rho_{g}}+\frac{1}{\rho_{g}}\nabla \times \bar{\tau }+\nabla p_{\mathrm{foam}}+g.$$

For Reynolds numbers higher than 4000, turbulence was computed using the *k-ε* turbulence model. Finally, for obtaining the temperature distribution in the porous matrix, the local thermal nonequilibrium (LTNE) model based on the volume averaging method is used. The LTNE model considers the energy equations for the fluid (Equation (11)) and solid phase (Equation (12)) [24,25,26] as

(11) $$G\left(P\frac{\partial T_{f}}{\partial t}+u_{g}\nabla \cdot T_{f}\right)=\nabla \cdot \left(k_{f}\cdot \nabla T_{f}\right)P+h_{g}\left(T_{s}-T_{f}\right)+PQ_{\mathrm{MW}}+{\rho_{d}M_{d}\delta_{d}\cdot H_{\mathrm{vap},d}|}_{\begin{array}{c} .\\ T_{f}=T_{\mathrm{boil}} \end{array}}\;$$

(12) $$\left(1-P\right)\rho_{s}C_{p,s}\frac{\partial T_{s}}{\partial t}+\left(1-P\right)\nabla \cdot \left(k_{s}\cdot \nabla T_{s}\right)+\left(1-P\right)Q_{\mathrm{MW}}=h_{g}\left(T_{s}-T_{f}\right)$$

where $C_{p}$ is the heat capacity, k is the heat conductivity, $h_{g}$ is the volumetric heat transfer coefficient (coupling Equations (11) and (12)), $T_{\mathrm{boil}}$, $M_{d}$ and $\cdot H_{\mathrm{vap},d}$ are boiling temperature, molar mass and enthalpy of evaporation of the droplets, respectively. Using a correlation developed by Dietrich [25], $h_{g}$ was calculated by incorporating the pressure drop in open-cell ceramic foams; the correlation is valid for $u_{g}$, ranging from 0.5 to 5 ${\mathrm{ms}}^{-1}$. For the energy equations, viscous dissipation and work done by pressure changes were assumed negligible. Moreover, an independent energy equation for the droplets was also not considered. Instead, it was considered to be in local thermal equilibrium (i.e., $T_{d}=T_{g})$ with the gas phase. Consequently, the terms of G and $k_{f}$ change according to $T_{f}$ as

(13) $$G=\{\begin{array}{c} \rho_{g}C_{p,g}\left(1-\delta_{d}\right)+\rho_{d}C_{p,d}\delta_{d}\\ \rho_{g}C_{p,g} \end{array}\begin{array}{c} \mathrm{for}\;\;\;T_{f}\leq T_{b}\\ \mathrm{for}\;\;\;T_{f}>T_{b} \end{array},$$

(14) $$k_{f}=\{\begin{array}{cc} k_{g}\frac{2\delta_{d}\left(k_{d}-k_{g}\right)+k_{d}+2k_{g}}{2k_{g}+k_{d}-\delta_{d}\left(k_{d}-k_{g}\right)} & \mathrm{for}\;\;\;T_{f}\leq T_{b}\\ k_{g} & \mathrm{for}\;\;\;T_{f}>T_{b} \end{array}.$$

As droplets are considered spherical, the Maxwell–Garnett mixing relation was used for estimating the heat conductivity of the fluid phase $k_{f}$.

### 2.5. Design of the Microwave Applicator

**Figure 3:**
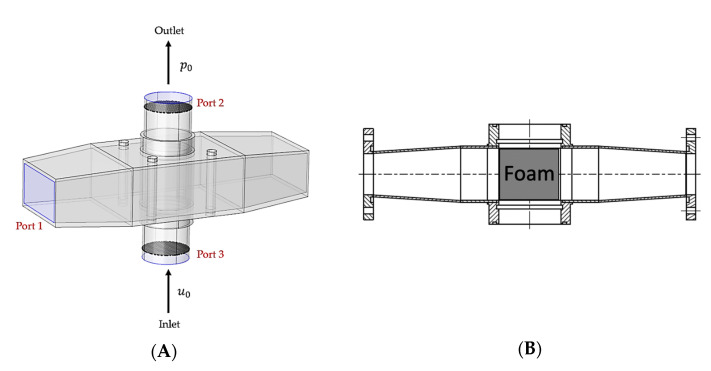
Microwave applicator design: (**A**) model design with boundary surfaces, (**B**) internal view plane displaying the foam position.

The governing equations and properties described above were used along with the finite element method in the commercial solver COMSOL Multiphysics (version 5.6, Comsol Inc., Stockholm, Sweden) to support the design of the microwave applicator and to study the droplet flow for which flow parameters were studied. The microwave applicator was designed to concentrate the power of a microwave source working at a frequency of 2.45 GHz to the ceramic foam. It consists of a rectangular waveguide WR430 with a cutoff frequency of 1.372 GHz and internal metallic rods. The droplet-laden stream crosses the applicator inside a quartz tube, as shown in Figure 3. The electric field is contained inside cavity and quartz tube by means of two metallic caps featuring woven wire meshes. Wire diameter and pore size of the mesh are 0.52 mm and 2.0 mm, respectively, which allows fluids to pass through the foam but confines microwaves within the applicator (due to the cutoff frequency of the mesh holes). Foams are fixed inside the quartz tube in between two tubes supporters, which are made of Polyetheretherketone (PEEK), which has good microwave transparency.

### 2.6. Mesh Size and Boundary Conditions

Boundary conditions applied in the numerical calculations are summarized in Table 2 and illustrated in Figure 3. Moreover, simulations with different mesh element size were performed to ensure grid-independent results. Eventually, a mesh with cell length segment ranging from 0.001 to 0.080 mm was chosen for computations.

### 2.7. Experimental Setup

**Figure 4:**
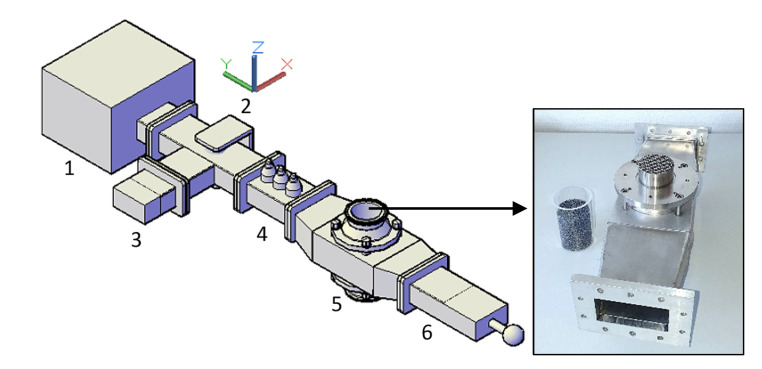
Configuration of the microwave applicator setup: (1) magnetron, (2) isolator, (3) water dummy load, (4) 3-stub tuner, (5) microwave applicator, (6) sliding short circuit. The microwave applicator is shown on the right with SiSiC ceramic foam sample and quartz tube.

For conducting experiments on droplet evaporation, the microwave applicator was constructed and equipped with a magnetron microwave generator, an isolator, a three-stub tuner for load impedance matching, a water dummy load and a sliding short circuit (see Figure 4). The latter system enables the modification of the electric field to preferred positions. Moreover, the tuner allows impedance matching of the magnetron to the microwave applicator. This was done by analyzing the voltage amplitude of the microwave signal at the dummy load using an oscilloscope (TDS3032B from Tektronix Inc., Beaverton, OR, USA). The magnetron (1200049 from Muegge GmbH, Reichelsheim, Hesse, Germany) operates at a frequency of 2.45 GHz with adjustable microwave power up to 2 kW (GEN2450/2.0KW2CSA from IBf electronic GmbH and Co., Ober-Ramstadt, Hesse, Germany).

**Figure 5:**
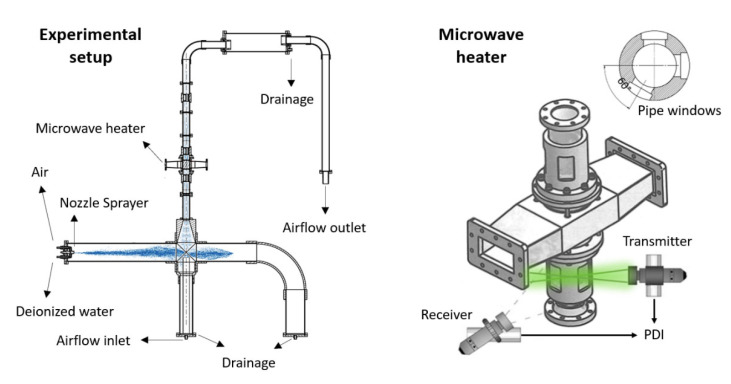
Experimental setup with details of microwave applicator and PDI measurement system.

Droplets are produced by an air-driven spray-nozzle atomizer (SU1A from Spraying Systems Co., Schorndorf, Baden-Wurtemberg, Germany) and are carried to the microwave applicator via humidified airflow (relative humidity *RH* = 85 ± 2%) provided from a packed column humidifier (see Figure 5, left). The airflow inlet velocity is adjusted via mass flow controller (FMA-2600A from Omega, Norwalk, CT, USA), providing a maximum liquid capacity of 2.2 L$h^{-1}$ for an atomizing air pressure of 4 bar. A 2D-phase Doppler interferometer (PDI, PDI-x00MD from Artium Technologies Inc., Sunnyvale, CA, USA) was used for measurements of droplet size, velocity, number density and flux at inlet and outlet of the microwave applicator.

PDI measurements were conducted at pipe components fabricated with rectangular windows. These windows were placed before and after the microwave applicator (see Figure 5, right) to measure the effects of foams and microwave heating on the entering droplet population. The exact measuring points were adjusted by intersecting the two laser beams from the PDI transmitter at 6 cm below (inlet position) and above (outlet position) the foam. Dry air (*RH* = 0%, $u_{g}=12{\;\mathrm{ms}}^{-1}$) was blown for 3 min before each measurement to remove any remaining water accumulation to avoid film formation or droplet production in pipelines and foams. Measurements from the PDI were performed at least three times per investigated parameter for each foam sample. To eliminate outliers, droplets that exceed more than three standard deviations of the average droplet size were removed. The input gas velocity and the magnetron input power were varied from 2.5 to 14.0 ${\mathrm{ms}}^{-1}$ and from 200 to 1000 W, respectively. A droplet flow with a volume fraction < 0.1 was carried to the microwave applicator. Experiments were performed under atmospheric pressure and controlled ambient temperature of 20 °C. Note that the microwave applicator is referred to as loaded when it contained a foam, otherwise is defined as unloaded.

## 3. Results and Discussion

### 3.1. Electric Field Distribution

**Figure 6:**
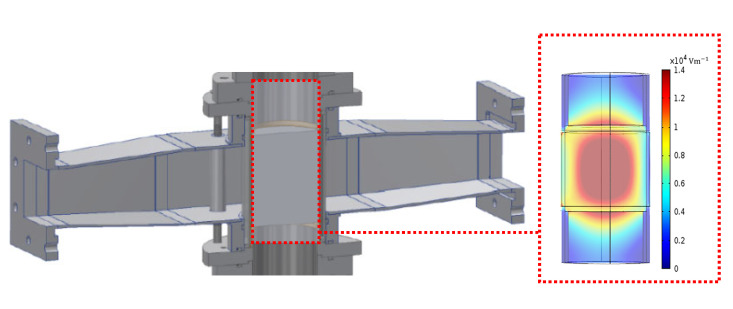
Example of the 3D maximum electric field distribution in the tube at the foam’s core (SBSiC, 30 ppi).

Numerical calculations were used to provide insight concerning the electric field distribution. Computations were performed using a wave excitation in the TE10 mode and a convergence criterion for the scaled residuals was set to 10^−4^. Figure 6 shows the electric field distribution in the quartz tube for the cases of tuning the system to maximize the electric field at the foam’s core by adjusting the sliding short circuit length. Maximum electric field strengths ranging from 14 to 30 k${\mathrm{Vm}}^{-1}$ (see Table 3) were achieved in the SiC-based foams for an input magnetron power of 1 kW. The cavity provides maximum heating to center of the foam’s core, as shown in Figure 6.

### 3.2. Microwave-Assisted Droplet Removal

**Figure 7:**
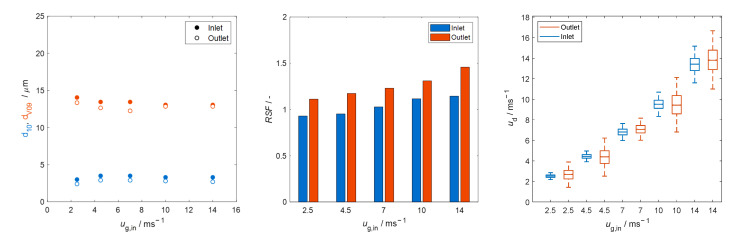
Diameter (**left**), relative span factor (**center**) and velocity (**right**) of the droplets at inlet and outlet of the unloaded microwave applicator.

The measured size and velocity of droplets at inlet and outlet positions of the unloaded (without foam) microwave applicator are shown in Figure 7. More than 20,000 counts (droplets) were considered for each measurement. Measurement data where the same parameters were used were cumulated into one, to report the results per parameter and not per measurement. Droplet size is reported in terms of $d_{10}$ (arithmetic mean diameter) and $d_{V09}$; the definition of the latter is that 90% of the total number of the sprayed volume is made of droplets with diameters smaller than or equal to $d_{V09}$. The uniformity of the droplet size distribution is quantified through the relative span factor $RSF=\left(d_{V09}-d_{V01}\right)/d_{V05}$, where $d_{V01}$ and $d_{V05}$ have the definition equivalent to $d_{V09}$ but accounting the 10% and 50%, respectively, of the total number of the sprayed volume.

**Figure 8:**
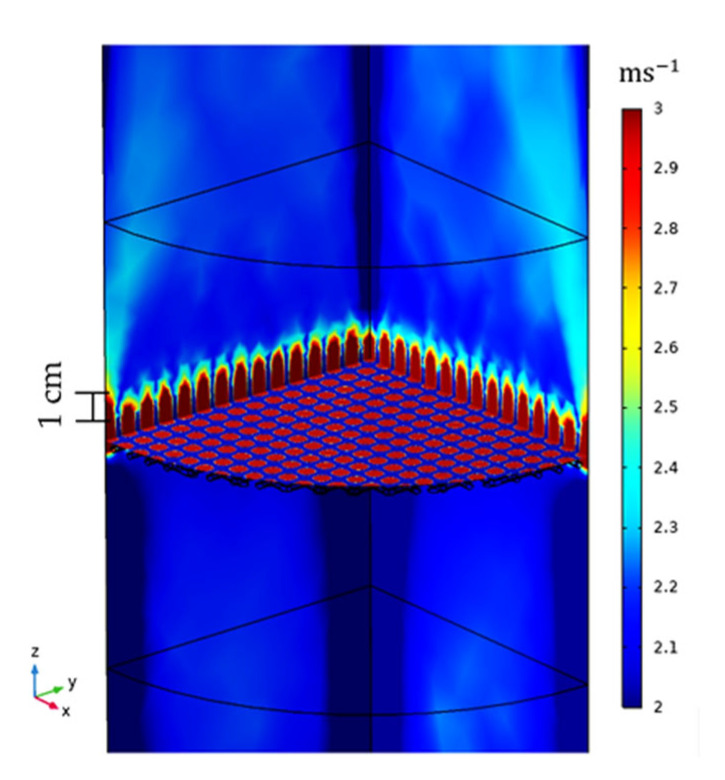
Examples of the gas velocity field for the nonheated unloaded microwave applicator using a 30 ppi foam and a gas flow in *z*-direction with an inlet velocity of $u_{g}=2.5{\;\mathrm{ms}}^{-1}$

Figure 7 (left) shows that the majority of primary droplets reaching the microwave applicator are smaller than 15 µm. Droplets at the inlet are completely entrained as their velocity equals to the mean gas velocity (see Figure 7, left). However, droplets crossing the outer position (located at 1 cm above the metallic mesh) are slightly faster than those from the inlet. The increase in the average velocity corresponds to the higher gas velocity developed due to a decrease in the cross-sectional area of the metallic wire mesh, which in accordance with numerical calculations prevails up to ~1 cm above the mesh, as shown in Figure 8.

.

Figure 7 (right) shows that the velocity distribution is wider at the outlet than at the inlet, because of a higher percentage of smaller droplets that produce a higher relative span factor, as seen in Figure 7 (center). The higher percentage of small droplets in the flow is caused by the reduction in droplet size, which—apart from being caused by droplet vaporization by diffusion—is also related to the surface filtering effect of the metallic wire mesh.

**Figure 9:**
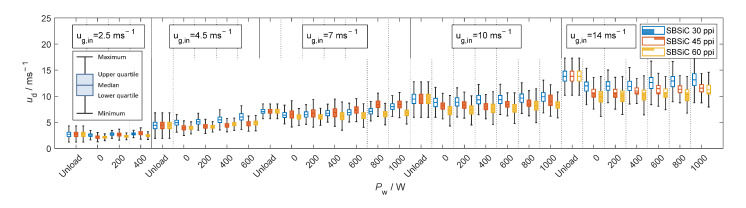

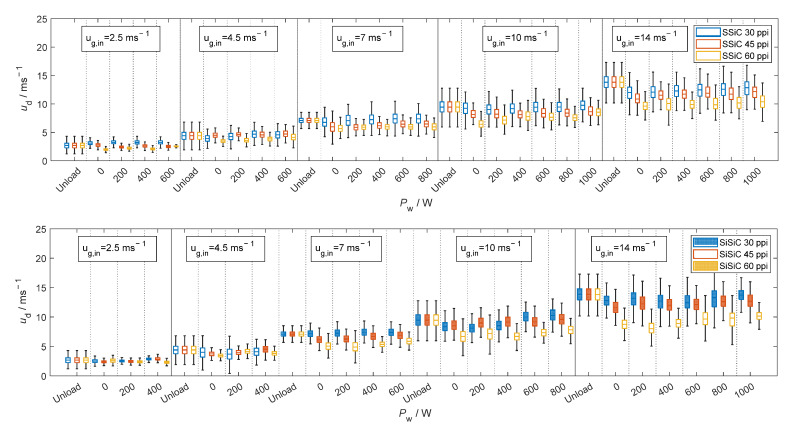
Velocity of droplets crossing the outlet position of the microwave applicator loaded with SBSiC (**top**), SSiC (**middle**) and SiSiC (**bottom**) foams at an input magnetron power up to 1 kW.

**Figure 10:**
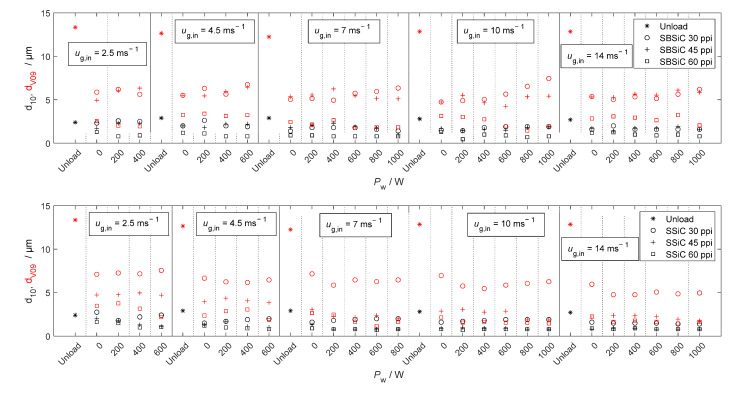

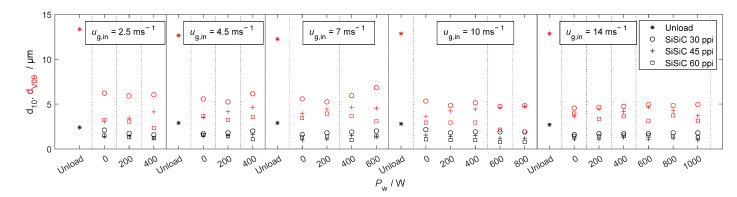
Diameters $d_{10}$ and $d_{V09}$ (red symbols) of droplets crossing the outlet position of the microwave applicator loaded with SBSiC (**top**), SSiC (**middle**) and SiSiC (**bottom**) foams at an input magnetron power up to 1 kW.

**Figure 11:**
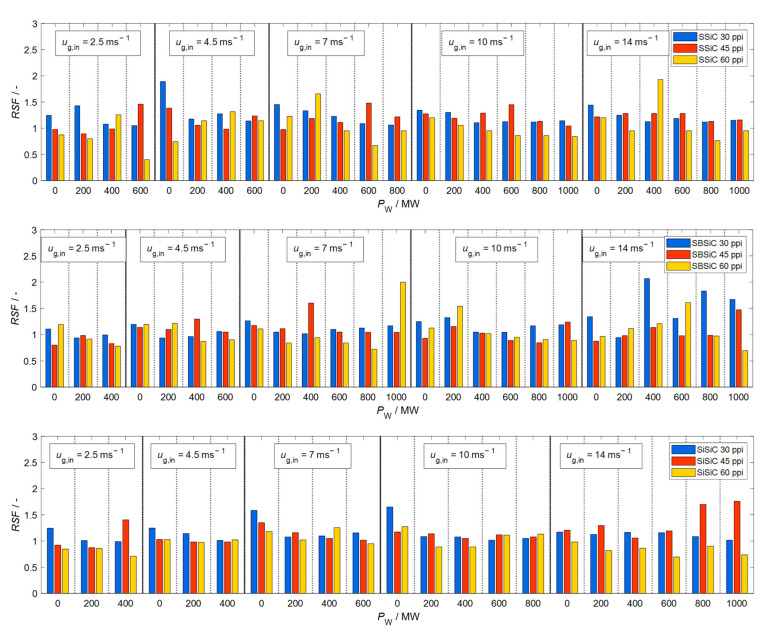
Relative span factor of droplets crossing the outlet position of the microwave applicator loaded with SBSiC (**top**), SSiC (**middle**) and SiSiC (**bottom**) foams at an input magnetron power up to 1 kW.

**Figure 12:**
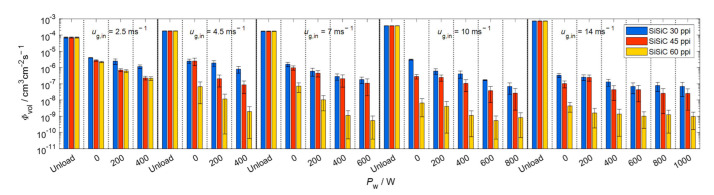

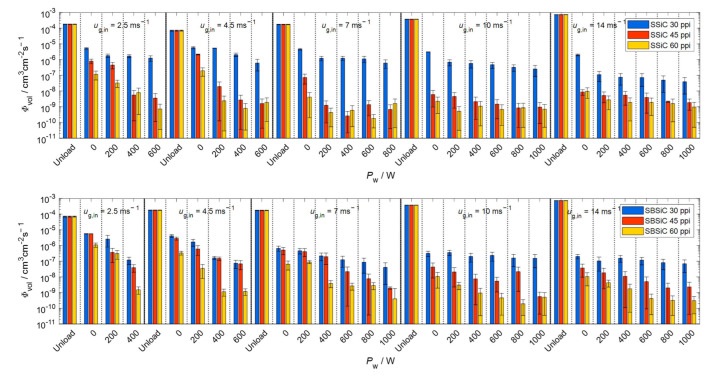
Volumetric flux of droplets crossing the outlet position of the microwave applicator loaded with SBSiC (**top**), SSiC (**middle**) and SiSiC (**bottom**) foams at input magnetron power up to 1 kW.

Figure 9, Figure 10, Figure 11 and Figure 12 show velocity, size, RSF and volumetric flux $\Phi_{\mathrm{vol}}$, respectively, of the droplets crossing the outlet position of the loaded (with SBSiC, SSiC and SiSiC foams) microwave applicator. Microwave heating cannot be applied to the unloaded cavity, since heating can only be applied if a microwave absorbing material is present. In order to avoid damage to the applicator due to the occurrence of arcing, the magnetron power is only increased at higher gas velocities. The arcing breakdown voltage decreases as the temperature rises due to a decrease in the air density [27]; thus, arcing does not occur at higher flow velocities that are further cooling the foams.

As shown in Figure 9, the average droplet velocity along the direction of the airflow decreases after impingement (i.e., rebounding and splashing) within the foams and thus decreases as the pore density increases. It should be noted that droplets have not regained the mean stream velocity at the outlet position. Additionally, as the input magnetron power increases the foam’s temperature increases while the gas density decreases. Therefore, both volumetric flow and gas velocity do also increase and entrained droplets move faster as the magnetron input power increases (see Figure 9).

Figure 10 shows that droplet size is reduced after passing through the loaded applicator, which is consistent with the well-known filtering effect of such foams. The size does not decrease as the input magnetron power increases. Thus, it can be concluded that most droplets are removed through interactions with the foams rather than evaporated by direct microwave radiation. This is also supported by the relative span factor shown in Figure 11, which does not show an increasing trend as the power increases. Given that the porosity of the foams is the same regardless of the pore density (see Table 1) and their effective permittivity is practically the same, then the microwave heating rate and the temperature should be similar for these foams. Therefore, the change in droplet size is caused by the filtering effect of the foams, as shown by the diameter, which decreases while the pore density increases.

Figure 12 shows that the volumetric flux of the droplets is mainly reduced by the filtering effect of the foams, since there is a reduction of up to three orders of magnitude in the volumetric flux of the droplets without microwave heating. While applying microwave heating, the volumetric flux decreases further by an order of magnitude. It should be noted that although clogging and water accumulation in foams was not explicitly quantified, no water accumulation was observed for input magnetron power ratings equal or above 400 W (even after 30 min of continuous operation), which reflects that the temperature in the foams was high enough to evaporate structure-borne liquid accumulations. In general, the volumetric flux is decreased according to the following order: SiSiC > SSiC > SBSiC. This order agrees with but reverse from the order of the maximum electric field strength within foams (see Table 3), which is consistent with an expected higher temperature and therefore higher evaporation rates.

## 4. Conclusions

In this study, we analyzed the potential of a compact technology based on capturing and in situ evaporation of droplets. The approach is based on heating open-cell foams by using microwaves. A microwave applicator was designed and the fluid characteristics of droplet-laden streams filtered through microwave-heated open-cell foams were studied. In accordance with numerical findings, the designed applicator concentrates the electric field on the open-cell foams, providing maximum heating strength to the flow’s core.

Size, velocity and volumetric flux of droplets under different gas velocities and different microwave-heated open-cell foams based on silicon carbide were measured. The main experimental conclusions from this work follow:

1. The volumetric flow of droplets is found to decrease as much as ~99.9% by using the open-cell foams as filter media, while microwave-heated foams result in a further reduction up to ~99.99%. The major contribution to droplet removal is due to mechanical filtration and not due to microwave selective heating.
2. Increasing the microwave input power causes a higher temperature in the open-cell foams, which in turn decreases the droplet volumetric flow via evaporation.
3. High temperatures in open-cell foams under microwave heating are found to prevent structure-borne liquid accumulations. Thus, the device presented has proven to be a compact solution for droplet removal in pipeline installations that combines primary and secondary droplet separation in a single step.
4. By evaporating structure-borne liquid accumulations, microwave heating is found to prevent water clogging in the fine droplet separator.

## Figures and Tables

**Table 1:** Porosity of applied SiC-based open-cell foams.

Pore Density /ppi	SBSiC			SSiC			SiSiC		
	30	45	60	30	45	60	30	45	60
P/-	0.902	0.905	0.906	0.896	0.896	0.903	0.868	0.87	0.874

**Table 2:** Boundary conditions applied for the numerical calculations.

Flow Boundary Conditions			
Interface	Velocity	Pressure	Remarks
Inlet	$\frac{du}{dx}=\frac{du}{dy}=\frac{du}{dz}=0$	Computed	$y^{+}$ is the dimensionless wall-thickness parameter
Outlet	$\frac{du}{dx}=\frac{du}{dy}=\frac{du}{dz}=0$	P=0	
Walls (no slip)	${u|}_{\left(y^{+}=0\right)}=0$	Computed	
**Electromagnetic Boundary Conditions**			
Interface	Electric Field		Remarks
Walls	n×E=0		Perfect electric conductor
Ports	Computed		
**Heat Transfer Boundary Conditions**			
Interface	Heat Flux		Remarks
Inlet	$-n\cdot q=\left(u\cdot n\right)\rho \int_{T_{\mathrm{inflow}}}^{T}C_{p}dT$		$T_{\mathrm{ext}}=T_{\mathrm{inflow}}=25\;{}^{\circ}C$ and n is the vector normal to the boundary
Outlet	−n·q=0		
Walls	$q=h\left(T_{\mathrm{ext}}-T\right)$		

**Table 3:** Maximum electric field strength in the foams for concentrating the electric field distribution to the foam’s core.

Foam	P/-	$\mathrm{Maximum}\;\mathrm{E-Field}/kV\cdot m^{-1}$
SBSiC	0.90	30
SSiC	0.90	25
SiSiC	0.87	14

## Data Availability

Data are contained within the article.

## Abbreviations

LTNE	Local thermal nonequilibrium
PEEK	Polyetheretherketone
PID	Phase Doppler interferometry
SBSiC	Silicon-bonded silicon carbide
SiSiC	Silicon infiltrated silicon carbide
SSiC	Pressureless sintered silicon carbide

## Nomenclature

a	Coefficients of the polynomials for calculating $q^{\prime }$ and $q^{\prime \prime }$
$C_{p}$	Heat capacity
d	Diameter
E	Electric field
g	Gravity acceleration constant
h	Volumetric heat transfer coefficient
Q	Heat rate
RH	Relative humidity
j	Complex number
k	Thermal conductivity
M	Molar mass
P	Porosity
RSF	Relative span factor
S	Specific surface area
*T*	Temperature
t	Time
u	Velocity
V	Volume domain
X	Inclusion-size parameter
Λ	Droplets residence time distribution
Φ	Flux
δ	Volume fraction
k,α,β,γ,ζ	Fitting parameters for a modified Dagum distribution
λ	Wavelength
q	Fitting geometrical parameter representing Platonic foams
$q^{\prime },q^{\prime \prime }$	Real and imaginary parts of *q*, respectively
ε	Complex permittivity
$\varepsilon^{\prime },\;\varepsilon^{\prime \prime }$	Dielectric constant and dielectric loss, respectively
μ	Dynamic viscosity
ρ	Density
ω	Angular frequency
τ	Rotational relaxation time of the molecules
$\bar{\tau }$	Summation of the viscous stress tensor and turbulence tensor
∇p	Pressure drop
ΔH	Enthalpy

## Subscripts

boil	Boiling
d	Droplets
eff	Effective
f	Medium filling the voids of open-cell foam
foam	Open-cell foam
g	Gas
in	Incident radiation
inlet	Position corresponding to the inlet
icl	Inclusion
m	Slope value of the linear expression of *q*
MW	Microwave
s	Solid
st	Static, equivalent to a wave with a frequency equal to zero
vap	Evaporation
vol	Volumetric
∞	Optical, equivalent to a wave with a frequency equal to infinite
V01, V05, V09	Total sprayed volume corresponding to 10%, 50% and 90%.
0	Ordinate-intercept value of the linear expression of *q*
